# FDG-PET/MR in paediatric pyrexia of unknown origin: early detection of Takayasu arteritis—a single-centre experience

**DOI:** 10.3389/fped.2026.1842712

**Published:** 2026-09-21

**Authors:** Ashwani Kumar, Suma Balan, Gopika P. Mohan

**Affiliations:** Department of Rheumatology and Clinical Immunology, Amrita Institute of Medical Sciences and Research Centre, Amrita Vishwa Vidyapeetham, Kochi, India

**Keywords:** childhood-onset Takayasu arteritis, early diagnosis in paediatric rheumatology, FDG-PET/MR, large-vessel vasculitis, pre-stenotic Takayasu arteritis, PUO

## Abstract

Pyrexia of unknown origin (PUO) in children presents a significant diagnostic challenge owing to its broad and heterogeneous aetiology. Among autoimmune causes, childhood-onset Takayasu arteritis (cTAK) is rare but clinically critical, typically characterised by non-specific constitutional symptoms and diagnosed late. Conventional angiographic imaging depicts structural vascular changes and may fail to identify the disease in its early, pre-stenotic inflammatory phase. We evaluated the role of 18F-fluorodeoxyglucose positron emission tomography/magnetic resonance imaging (^18^F-FDG-PET/MR) in the early detection of pre-stenotic cTAK in children presenting with PUO and its impact on management and short-term outcomes. In this single-centre, retrospective descriptive case series conducted over 2 years, children (≤18 years) presenting with PUO who underwent ^18^F-FDG-PET/MR and were subsequently diagnosed with pre-stenotic Takayasu arteritis (paediatric European League Against Rheumatism/Paediatric Rheumatology International Trials Organisation/Paediatric Rheumatology European Society 2008 criteria) were analysed. During the study period, 28 children with PUO underwent ^18^F-FDG-PET/MR; 7 (25%) were diagnosed with pre-stenotic cTAK (mean age 13.9 years; 5 girls), and of the remaining 21, 20 received alternative diagnoses. All had constitutional symptoms and markedly elevated inflammatory markers (mean ESR of 104 mm/h; mean C-reactive protein of 128.4 mg/L), with infrequent overt vascular signs. ^18^F-FDG-PET/MR demonstrated FDG-avid wall thickening of the aorta and its major branches in every patient (maximum standardised uptake value 2.7–7.4; median 4.8), without haemodynamically significant stenosis at baseline. All children received pulsed methylprednisolone, oral prednisolone, and mycophenolate mofetil; three required tocilizumab and one adalimumab. Over a median follow-up of 20 months (range 9–36), one child developed a short-segment left subclavian stenosis, while the remainder showed no new structural progression, occlusion, or pulseless features. ^18^F-FDG-PET/MR detected metabolically active large-vessel inflammation in the pre-stenotic phase, enabling early diagnosis and treatment. These preliminary, hypothesis-generating findings support further investigation of ^18^F-FDG-PET/MR in selected children with PUO and persistent inflammation; prospective, multicentre validation with standardised paediatric protocols and longer follow-up is required before routine integration into diagnostic pathways.

## 1 Introduction and review of literature

Pyrexia of unknown origin (PUO) remains one of the most challenging diagnostic scenarios in paediatric medicine. It was classically defined by Petersdorf and Beeson in 1961 as a temperature greater than 38.3 °C on several occasions, accompanied by more than 3 weeks of illness, with failure to reach a diagnosis after 1 week of inpatient investigation (1). Modern paediatric definitions adopt a more flexible timeframe, i.e., fever of ≥38.3 °C, occurring at least once daily for ≥8 days, with an inconclusive history, physical examination, and preliminary investigations assessed on an outpatient basis (2).

PUO accounts for a notable proportion of paediatric admissions, with considerable variation across settings that reflects the local infectious-disease burden (2). Its aetiological spectrum is broad and heterogeneous: in a systematic review of paediatric PUO, infections were the leading identifiable cause (∼51%), followed by miscellaneous non-infectious conditions (∼11%), collagen-vascular/autoimmune disease including vasculitis (≈9%), and malignancy (∼6%), while a substantial proportion (∼23%) remained undiagnosed (2). Among the autoimmune causes, large-vessel vasculitis—particularly Takayasu arteritis (TAK)—is a rare but clinically critical diagnosis that is frequently overlooked or delayed because of its insidious onset and non-specific early manifestations (3, 4).

Takayasu arteritis is a chronic granulomatous large-vessel vasculitis of unknown aetiology that characteristically affects the aorta and its major branches, including the brachiocephalic, carotid, subclavian, renal, and mesenteric arteries (5, 6). It predominantly affects young women, with the majority of cases occurring between the ages of 20 and 40 years. However, approximately 20% of cases begin in childhood [childhood-onset TAK (cTAK)] (3). Recent meta-analytic data indicate an incidence of 0.3–3.3 per million person-years, with markedly higher prevalence in Asia (up to 360 per million in selected populations) (7). In the paediatric population, estimates are substantially lower and vary by region; nationwide data from South Korea, for example, describe a prevalence rising from 0.09 per 100,000 in children aged 0–4 years to 0.80 per 100,000 in those aged 15–19 years (8). Whereas adult TAK shows a strong female predominance (up to ∼90%), the female preponderance in cTAK is lower, estimated at 2.5:1 to 3:1, and the mean age at onset is approximately 12 years, although onset has been documented across all paediatric ages (3).

The clinical presentation of cTAK is notoriously non-specific and insidious. Children typically present with constitutional symptoms—fever, malaise, weight loss, and fatigue—and may conspicuously lack the classical vascular stigmata of advanced disease, such as absent pulses, inter-arm blood-pressure discrepancy, carotid bruits, and claudication (3, 4). Since Takayasu's original description of the characteristic retinal and pulse findings in 1908 (9), recognition has often depended on these late features, and diagnostic delay is consequently common in children, with some series reporting intervals of several months to more than a year (4). This delay is consequential: during the unrecognised period, the underlying granulomatous vasculitis progresses, producing irreversible vascular remodelling—vessel-wall thickening, intimal proliferation, and ultimately fixed stenosis, occlusion, or aneurysmal dilatation (10). Early immunosuppressive intervention during the active inflammatory phase has been linked to prevention or mitigation of these complications, suggesting that timely diagnosis is prognostically critical, although the supporting paediatric evidence remains limited (11).

Conventional structural imaging—magnetic resonance angiography (MRA), computed tomography angiography (CTA), and Doppler ultrasonography—depicts the anatomical consequences of vascular disease, such as luminal narrowing, wall thickening, dilatation, and calcification. These modalities reliably detect established remodelling but cannot consistently differentiate active inflammatory disease from chronic fibrotic change nor reliably identify inflammation in the “pre-stenotic” phase, where active vascular wall inflammation exists without haemodynamically significant luminal alteration (12, 13). In this phase, angiographic imaging may appear normal despite ongoing granulomatous inflammation of the arterial wall, limiting the utility of structural imaging in the earliest and most therapeutically responsive stage of disease.

Positron emission tomography with ^18^F-fluorodeoxyglucose (FDG-PET) offers a fundamentally distinct and complementary approach that focuses on metabolic activity rather than anatomical changes. ^18^F-FDG, a glucose analogue, is taken up preferentially by metabolically active inflammatory cells infiltrating the arterial wall (14). Activated macrophages and lymphocytes within the adventitia, media, and intima of affected vessels have a high metabolic demand and upregulate glucose transporters, particularly GLUT1 and GLUT3, which facilitate glucose uptake into cells (15). Once transported intracellularly, ^1^⁸F-FDG is phosphorylated by hexokinase to FDG-6-phosphate, which is trapped within cells and cannot be metabolised further or exported (14). Consequently, the quantity of accumulated ^18^F-FDG is proportional to the metabolic activity of the inflammatory infiltrate, enabling quantification of vascular inflammation independent of luminal diameter or remodelling (14).

Because FDG uptake reflects inflammatory cell burden rather than luminal calibre, FDG-PET can detect active inflammation during the pre-stenotic phase, when the vessel wall is densely infiltrated before luminal narrowing develops—identifying disease while it remains therapeutically responsive and before irreversible damage occurs (12). The pattern and distribution of large-vessel uptake further help to characterise the extent of arterial involvement (16). Arterial wall FDG uptake equal to or exceeding hepatic activity has become an accepted criterion for active large-vessel vasculitis, and the maximum standardised uptake value (SUVmax) provides a reproducible quantitative measure of disease activity (12, 17).

Despite extensive validation in adults, paediatric data on FDG-PET for vasculitis remain limited. The paediatric evidence base consists largely of small retrospective series, case reports, and meta-analyses that are primarily focused on adult patients. The largest prospective paediatric study, by Pijl et al., evaluated 110 children with PUO and reported an overall diagnostic yield of 48% (sensitivity 85.5%, specificity 79.2%); however, it included only two cases of TAK, providing little TAK-specific information (18). Paediatric-specific considerations—radiation exposure (a weight-adjusted effective dose of approximately 3–5 mSv with current dosimetry), technical feasibility in younger children, protocol standardisation, and cost—have further constrained both adoption and study of PET in paediatric rheumatological conditions (18).

At the guideline level, the European Association of Nuclear Medicine (EANM) 2024 consensus on [^18^F]FDG-PET/CT in fever and inflammation of unknown origin recognised a definite role for FDG-PET/CT in the diagnostic pathway, although it drew predominantly on adult data (19). Likewise, the 2023 update of the European League Against Rheumatism (EULAR) recommendations on imaging in large-vessel vasculitis accepts FDG-PET/CT as an alternative to MRA, while still recommending MRA as the preferred first-line modality in paediatric patients owing to its specificity, morphological resolution, and absence of ionising radiation (20).

A substantial gap therefore remains. Although FDG-PET/CT has established diagnostic efficacy in adult large-vessel vasculitis and demonstrable value in paediatric PUO, dedicated data on FDG-PET/CT or PET/MR for the early detection of cTAK presenting as PUO are scarce, and no large paediatric cohort has formally characterised the vascular involvement patterns or clinical implications in this setting. This is clinically important because identifying TAK during the pre-stenotic phase could enable timely immunosuppression and potentially avert irreversible vascular complications that strongly influence long-term morbidity and mortality. The present descriptive case series addresses this gap by evaluating FDG-PET/MR in a small group of children who presented with PUO and were subsequently diagnosed with pre-stenotic TAK, with particular attention paid to imaging findings that precede structural change and their impact on diagnostic timing and vascular outcomes.

## 2 Materials and methods

This is a single-centre, descriptive case series conducted at the Department of Rheumatology, Amrita Institute of Medical Sciences, Kochi, Kerala, India, over a 2-year period (March 2023–March 2025). All clinical, laboratory, and imaging data were retrieved from a prospectively maintained departmental database and the electronic medical record. The study was approved by the Institutional Ethics Committee.

### 2.1 Patient selection

Children aged ≤18 years at presentation were eligible if they (i) had presented with PUO, (ii) had undergone FDG-PET/MR as part of the PUO work-up, and (iii) were subsequently diagnosed with pre-stenotic TAK. PUO was defined as a documented fever of ≥38.3 °C on at least one occasion persisting for ≥3 weeks (or ≥8 days of in-hospital evaluation) without a diagnosis after an appropriate initial clinical, laboratory, and imaging work-up by the primary team (2).

^18^F-FDG-PET/MR was not performed routinely in all children with PUO; rather, it was used selectively in children with PUO that persisted despite a standard work-up, with inflammatory markers disproportionately elevated to any infective findings and clinical suspicion of an underlying inflammatory or autoimmune aetiology. This selective application is acknowledged as a source of spectrum bias in Section 4, as the cohort is thereby enriched for a phenotype likely to yield positive PET findings.

The diagnosis of TAK required fulfilment of the 2008 EULAR/Paediatric Rheumatology International Trials Organisation/Paediatric Rheumatology European Society (PRINTO/PRES) criteria for childhood TAK (21), which require an angiographic abnormality of the aorta or its main branches—including arterial wall thickening—together with at least one of hypertension, pulse deficit, or claudication; a four-limb blood-pressure discrepancy; a bruit; or raised acute-phase reactants, applied after the exclusion of conditions that mimic large-vessel vasculitis. The 2022 American College of Rheumatology (ACR)/EULAR classification criteria for Takayasu arteritis were derived from the Diagnostic and Classification Criteria for Vasculitis (DCVAS) cohort, which only enrolled patients aged ≥18 years and is therefore liable to miss the early, pre-stenotic presentations that characterise childhood-onset disease (22). The clinical, laboratory, and imaging features contributing to classification for each child are reported in Table 1.

**Table 1:** Individual patient-level data for the seven children with pre-stenotic childhood-onset Takayasu arteritis.

Parameter	P1	P2	P3	P4	P5	P6	P7	Summary (*n* = 7)	IQR (Q1–Q3)
Demographics									
Age (years)	17	16	14	11	11	11	17	**13.9, 14 (11–17)**	**11**–**16.5**
Sex	M	F	F	M	F	F	F	**5 F/2 M**	
Symptom/PUO duration (months)	2	3	3	3	<1	<1	5	**3 (<1–5)**	**—**
Constitutional symptoms									
Fever	Y	Y	Y	Y	Y	Y	N	**6**/**7**	
Weight loss	N	Y	Y	N	N	Y	N	**3**/**7**	
Fatigue	Y	Y	N	N	Y	Y	Y	**5**/**7**	
Arthralgia	Y	N	Y	Y	N	N	N	**3**/**7**	
Myalgia	N	N	N	N	N	N	Y	**1**/**7**	
Vascular/cardiovascular features									
Hypertension	N	Y	N	N	N	N	N	**1**/**7**	
Claudication	Y	N	Y	N	N	N	N	**2**/**7**	
Decreased/absent peripheral pulses	N	N	Y	N	N	N	N	**1**/**7**	
Inter-arm systolic BP difference >20 mmHg	N	Y	N	Y	N	N	N	**2**/**7**	
Arterial bruit	N	N	N	N	N	N	N	**0**/**7**	
Carotid artery abnormality	N	N	N	N	N	N	N	**0**/**7**	
Pulmonary arterial hypertension	N	N	N	Y	N	N	N	**1**/**7**	
CNS involvement	N	Y	N	Y	N	N	N	**2**/**7**	
Laboratory (baseline)									
ESR (mm/h)	116	119	103	90	110	81	109	**104, 109 (81–119)**	**96**–**113**
CRP (mg/L)	224	204	48	105	140	65	113	**128.4, 113 (48–224)**	**85**–**172**
Haemoglobin (g/dL)	11.3	9.5	11.6	10.6	10.1	12.9	10.8	**11.0, 10.8 (9.5–12.9)**	**10.4**–**11.5**
TLC (×10^3^/µL)	10.6	22.9	9.99	9.65	7.42	12.54	8.07	**11.6, 9.99 (7.4–22.9)**	**8.9**–**11.6**
Platelets (×10^3^/µL)	838	560	402	508	439	432	486	**523.6, 486 (402–838)**	**436**–**534**
Albumin (g/dL)	3.1	3.5	4.2	4.1	3.9	4.2	4.1	**3.9, 4.1 (3.1–4.2)**	**3.7**–**4.2**
Globulin (g/dL)	5.58	4.58	4.15	4.81	4.16	4.1	4.02	**4.5, 4.16 (4.02–5.58)**	**4.13**–**4.70**
Creatinine (mg/dL)	0.59	0.48	0.51	0.61	0.49	0.44	0.55	**0.52, 0.51 (0.44–0.61)**	**0.49**–**0.57**
FDG-PET/MR imaging									
SUVmax (most active segment)	4.6	4.8	2.7	3.7	6.8	7.4	5.3	**5.0, 4.8 (2.7–7.4)**	**4.2**–**6.0**
Aortic involvement (thoracic/abdominal)	Both	Both	Abd	Abd	Both	Thor	Both	**7**/**7**	
Disease activity									
ITAS-2010 (baseline)	6	6	8	3	2	1	2	**4.0, 3 (1–8)**	**2**–**6**
Treatment									
IV methylprednisolone	Y	Y	Y	Y	Y	Y	Y	**7**/**7**	
Oral prednisolone	Y	Y	Y	Y	Y	Y	Y	**7**/**7**	
Mycophenolate mofetil	Y	Y	Y	Y	Y	Y	Y	**7**/**7**	
Tocilizumab	Y	Y	N	N	N	Y	N	**3**/**7**	
Adalimumab (TNF inhibitor)	Y	N	N	N	N	N	N	**1**/**7**	
Follow-up									
Follow-up duration (months)	36	36	9	30	20	16	12	**22.7; 20 (9–36)**	
Repeat vascular imaging	Yes	Yes	No	No	Yes	No	No	**3**/**7**	
New stenosis on follow-up	No	No	—	—	Yes^a^	—	—	**1**/**7**	

Bold values denote cohort-level summary statistics (Summary and IQR columns) as distinct from individual patient data.

Continuous variables are summarised as mean; median (range), with the interquartile range (IQR, Q1–Q3) shown separately; categorical variables are reported as the number of patients with the feature (n/7). All index studies were ^18^F-FDG-PET/MR and were performed before glucocorticoid initiation. Thor, thoracic aorta; Abd, abdominal aorta; ESR, erythrocyte sedimentation rate; CRP, C-reactive protein; TLC, total leukocyte count; SUVmax, maximum standardised uptake value; ITAS-2010, Indian Takayasu Clinical Activity Score; IQR, interquartile range; CNS, central nervous system; TNF, tumour necrosis factor.

a New short-segment left subclavian stenosis in Patient 5 at 17 months.

Pre-stenotic TAK at baseline was defined as active large-vessel inflammation on ^18^F-FDG-PET/MR without haemodynamically significant luminal stenosis (defined *a priori* as >50% diameter narrowing), occlusion, or established aneurysm on baseline MR angiography. The >50% threshold was adopted as the conventional criterion for flow-limiting stenosis in cross-sectional and catheter angiography and is consistent with current EULAR imaging recommendations (20). Circumferential mural thickening without >50% narrowing did not, by itself, qualify as stenotic disease but was recorded as a clinically and pathologically significant early structural marker of arterial wall inflammation that reinforced the diagnosis of pre-stenotic TAK in conjunction with the PET findings.

Children with established fixed stenotic or occlusive (“pulseless”) TAK at first presentation, those who did not meet PUO criteria, and those in whom PET was performed primarily for an indication other than PUO were excluded. During the study period, 28 consecutive children with PUO underwent ^18^F-FDG-PET/MR and were identified from the departmental database; their final diagnoses were recorded. Seven were diagnosed with pre-stenotic Takayasu arteritis and constitute the analytic cohort; the remaining 21 received alternative final diagnoses (Table 2). Two further children with childhood-onset Takayasu arteritis diagnosed without FDG-PET (on MR angiography), one of whom lacked a documented fever, were not part of this PET/MR denominator. Because all 28 children were enrolled by virtue of having undergone ^18^F-FDG-PET/MR, and the diagnosis of pre-stenotic Takayasu arteritis itself incorporated the PET/MR finding, formal diagnostic accuracy metrics (sensitivity, specificity, and predictive values) for TAK were not calculated, as this would be subject to incorporation bias; the denominator instead permits the diagnostic yield of ^18^F-FDG-PET/MR in this selected PUO population to be reported descriptively, and the findings are regarded as hypothesis-generating.

**Table 2:** Diagnostic spectrum of the 28 children with pyrexia of unknown origin who underwent ^18^F-FDG-PET/MR during the study period.

Diagnostic category	n	% of 28
*Large-vessel vasculitis (Takayasu arteritis)*	7	25.0%
Autoimmune/autoinflammatory	7	25.0%
Malignancy	4	14.3%
Histiocytosis	4	14.3%
Infection	4	14.3%
Nutritional (scurvy)	1	3.6%
Undiagnosed	1	3.6%
*Total*	**28**	**100%**

A final diagnosis was established in 27 of 28 (96.4%); one child remained undiagnosed. Takayasu arteritis accounted for 7 (25.0%).

Bold values denote column totals.

### 2.2 Definitions

*Pre-stenotic TAK*: Imaging evidence of large-vessel wall inflammation on ^18^F-FDG-PET/MR and/or circumferential wall thickening, without haemodynamically significant luminal stenosis (>50% narrowing), occlusion, or aneurysm at baseline.

*Constitutional symptoms*: Documented fever, weight loss, fatigue, arthralgia, and/or myalgia at or before the initial rheumatology evaluation.

*Cardiovascular involvement*: Presence at baseline of any of hypertension (blood pressure ≥95th percentile for age, sex, and height, or a requirement for antihypertensive therapy), angina or exertional chest discomfort, claudication, arterial bruit, decreased or absent peripheral pulses, an inter-arm systolic blood pressure difference >20 mmHg, carotid artery abnormality, or pulmonary arterial hypertension documented on echocardiography.

*Central nervous system involvement*: New-onset headache, light-headedness, visual disturbance, seizures, focal neurological deficit, or stroke attributed to vasculitis.

*Disease activity* was assessed using the Indian Takayasu Clinical Activity Score (ITAS-2010), a validated 44-item instrument that scores new or worsening clinical features attributable to active vascular disease over the preceding ∼3 months across cardiovascular, neurological, musculoskeletal, and constitutional domains, with a score ≥2 generally indicating active disease (23). ITAS-2010 was documented at baseline in all seven patients. The instrument was chosen because it was developed and validated in an Indian population (including paediatric patients) comparable to our cohort, making its use internally consistent; its limited international adoption is acknowledged as a constraint on cross-study comparability, and a standardised tool is recommended for future multicentre studies.

### 2.3 FDG-PET/MR acquisition and interpretation

All the patients underwent whole-body ^18^F-FDG-PET/MR, performed according to the institutional protocol. The patients fasted overnight for at least 8 h, and venous blood glucose was confirmed to be within acceptable limits (≤130 mg/dL) before tracer administration. A weight-adjusted activity of ∼3.3 MBq/kg ^18^F-FDG was administered intravenously in accordance with EANM paediatric dosimetry guidance. After an uptake period of 60 min, whole-body simultaneous PET/MR imaging (vertex to feet) was acquired on a Siemens Biograph mMR (Siemens Healthineers, Erlangen, Germany), a high-definition PET system with lutetium oxyorthosilicate (LSO) crystals integrated with 3T MRI, using the syngo MR E11 platform (Siemens Healthineers, Erlangen, Germany). PET images were co-registered with MR images for interpretation.

In all seven patients, ^18^F-FDG-PET/MR was performed before the initiation of glucocorticoid therapy. Consequently, tracer uptake was not attenuated by immunosuppression—a recognised confounder that can rapidly suppress the vascular FDG signal and reduce diagnostic sensitivity (24)—which strengthens the interpretation of the imaging findings in this series.

SUVmax was recorded from the most metabolically active vascular segment using a volume of interest drawn on the fused images, and vascular uptake was graded visually relative to hepatic background. A scan was classified as consistent with large-vessel vasculitis when arterial wall uptake equalled or exceeded hepatic activity along the aorta and/or its major branches, in the absence of an alternative non-vasculitic explanation, in keeping with the joint EANM/SNMMI procedural recommendation (17). PET studies were reported by the nuclear medicine service as part of routine clinical care; because this is a retrospective case series in which large-vessel vasculitis was among the differential diagnoses stated at the time of referral, reporting was not blinded to the clinical suspicion—a limitation inherent to the retrospective design.

Involved vascular territories were grouped as thoracic aorta, abdominal aorta, subclavian arteries, pulmonary arteries, renal arteries, mesenteric arteries, and other territories (for example, the brachiocephalic trunk, common carotid, vertebral, and iliac or femoral arteries).

Alternative causes of large-vessel FDG uptake were systematically excluded. All patients underwent an infective work-up comprising blood cultures, viral serology, serology for organisms capable of causing infective aortitis (including *Brucella* and *Salmonella*), and tuberculosis evaluation (Mantoux test and interferon-*γ* release assay, with polymerase chain reaction where indicated). Malignancy was screened for using the whole-body FDG-PET metabolic survey, supplemented by bone-marrow examination where constitutional symptoms were prominent; and an autoimmune panel was performed. No alternative infective, neoplastic, or autoimmune cause was identified in any patient.

Continuous variables are summarised as mean and as median (range), with the interquartile range (Q1–Q3) reported separately. Individual patient-level data are presented in full to allow appraisal of the variability inherent in this small series.

## 3 Results

Over a 2-year period (March 2023–March 2025), 28 children presenting with PUO underwent ^18^F-FDG-PET/MR at our centre. A final diagnosis was established in 27 of the 28 (96.4%); 1 child remained undiagnosed. Seven children (25.0%) were diagnosed with pre-stenotic childhood-onset Takayasu arteritis and constitute the analytic cohort of this series. Two further children with childhood-onset Takayasu arteritis were diagnosed during the period without FDG-PET (on cross-sectional MR angiography), one of whom did not fulfil PUO criteria owing to the absence of documented fever, and are not included.

The remaining 21 children who underwent ^18^F-FDG-PET/MR received a range of alternative diagnoses: other autoimmune or autoinflammatory disease in 7 (systemic juvenile idiopathic arthritis, Kikuchi disease, periodic fever syndrome, and inflammatory bowel disease), malignancy in 4 (B-cell acute lymphoblastic leukaemia, Ewing sarcoma, ganglioneuroblastoma, and metastatic neuroblastoma), histiocytosis in 4 (Langerhans cell histiocytosis and Erdheim–Chester disease), and infection in 4 (disseminated methicillin-sensitive *Staphylococcus aureus* infection, tuberculosis, and brucellosis), with 1 case of scurvy (Table 2). The median age of the 28 children was 11.3 years (range 1.1–17.3); all 7 children with Takayasu arteritis were ≥11 years of age, whereas malignancy, histiocytosis, and infection predominated in younger children.

The seven children (five girls, two boys) had a mean age of 13.9 years (range 11–17). The median duration of symptoms before diagnosis was 3 months (range <1–5 months).

### 3.1 Clinical features at presentation

Constitutional symptoms dominated the presentation. Fever was documented in six of the seven children, and the remaining child presented with prolonged fatigue and myalgia; weight loss was present in three, fatigue in five, and musculoskeletal symptoms (arthralgia and/or myalgia) in four. Overt vascular signs were infrequent at baseline. One child had age-adjusted hypertension, and two reported limb claudication, accompanied by reduced peripheral pulses in one; two children had an inter-arm systolic blood pressure difference exceeding 20 mmHg. None had angina, audible arterial bruits, or clinically apparent carotid abnormalities. Two children experienced transient central nervous system symptoms (light-headedness) without focal neurological deficit, and one had pulmonary arterial hypertension on echocardiography.

### 3.2 Laboratory findings

Laboratory parameters reflected marked systemic inflammation. The mean erythrocyte sedimentation rate (ESR) was 104 mm/h (range 81–119 mm/h) and the mean C-reactive protein (CRP) 128.4 mg/L (range 48–224). There was mild normocytic anaemia (mean haemoglobin 11.0 g/dL) and reactive thrombocytosis (mean platelet count 523.6 × 10^3^/µL; mean total leukocyte count 11.6 × 10^3^/µL). Serum proteins showed an inflammatory pattern (mean albumin 3.9 g/dL, mean globulin 4.5 g/dL). Renal function and urinalysis were normal in all children (mean creatinine 0.52 mg/dL). The Indian Takayasu Clinical Activity Score (ITAS-2010) was documented at baseline in all seven children, with a median of 3 (mean 4.0; range 1–8) (Table 1).

### 3.3 FDG-PET/MR findings

All seven children underwent ^18^F-FDG-PET/MR, and every study was performed before the initiation of glucocorticoid therapy. FDG-avid wall thickening of the thoracic and/or abdominal aorta was present in all patients (thoracic aorta 5/7; abdominal aorta 6/7; aortic involvement 7/7). The next most frequently involved territories were the mesenteric arteries (4/7) and the subclavian and pulmonary arteries (3/7 each), followed by the renal arteries (2/7). Additional territories involved in individual patients included the common and internal carotid arteries; the brachiocephalic trunk; the celiac artery; and the iliofemoral, axillary, and popliteal vessels (Figure 1). Uptake ranged from subtle focal to diffuse circumferential wall involvement, with the SUVmax in the most active segment ranging from 2.7 to 7.4 (median 4.8). On the co-registered MR angiographic component, mural thickening was evident, but there was no haemodynamically significant (>50%) luminal stenosis, occlusion, or aneurysm in any patient at baseline; a representative study is shown in Figure 2.

**Figure 1:**
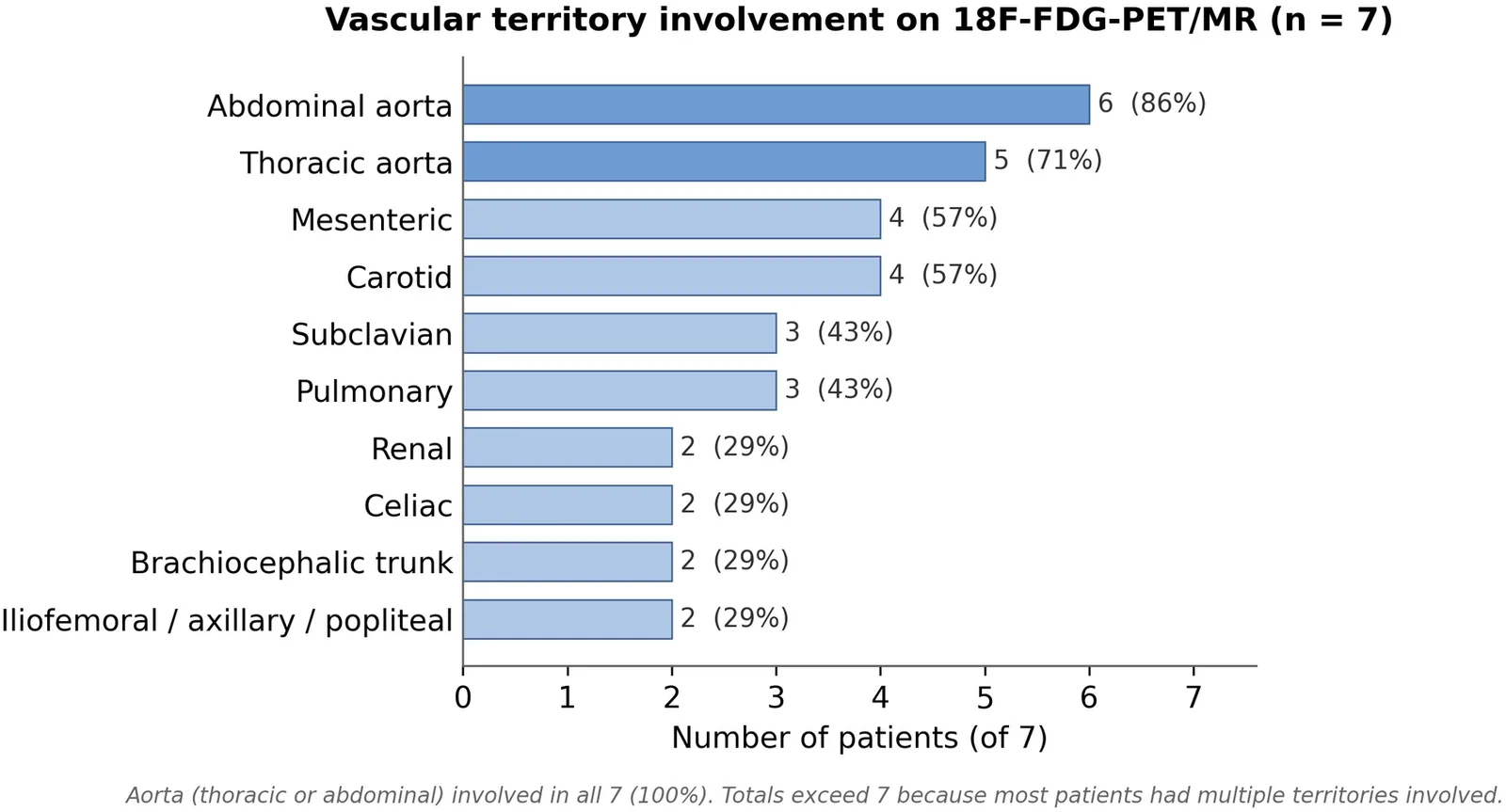
Vascular territories involved on ^18^F-FDG-PET/MR (*n* = 7). Bars show the number (and percentage) of patients with FDG-avid involvement of each territory. The thoracic or abdominal aorta was involved in all seven patients (100%). Totals exceed seven because the majority of the patients had involvement of multiple territories.

**Figure 2:**
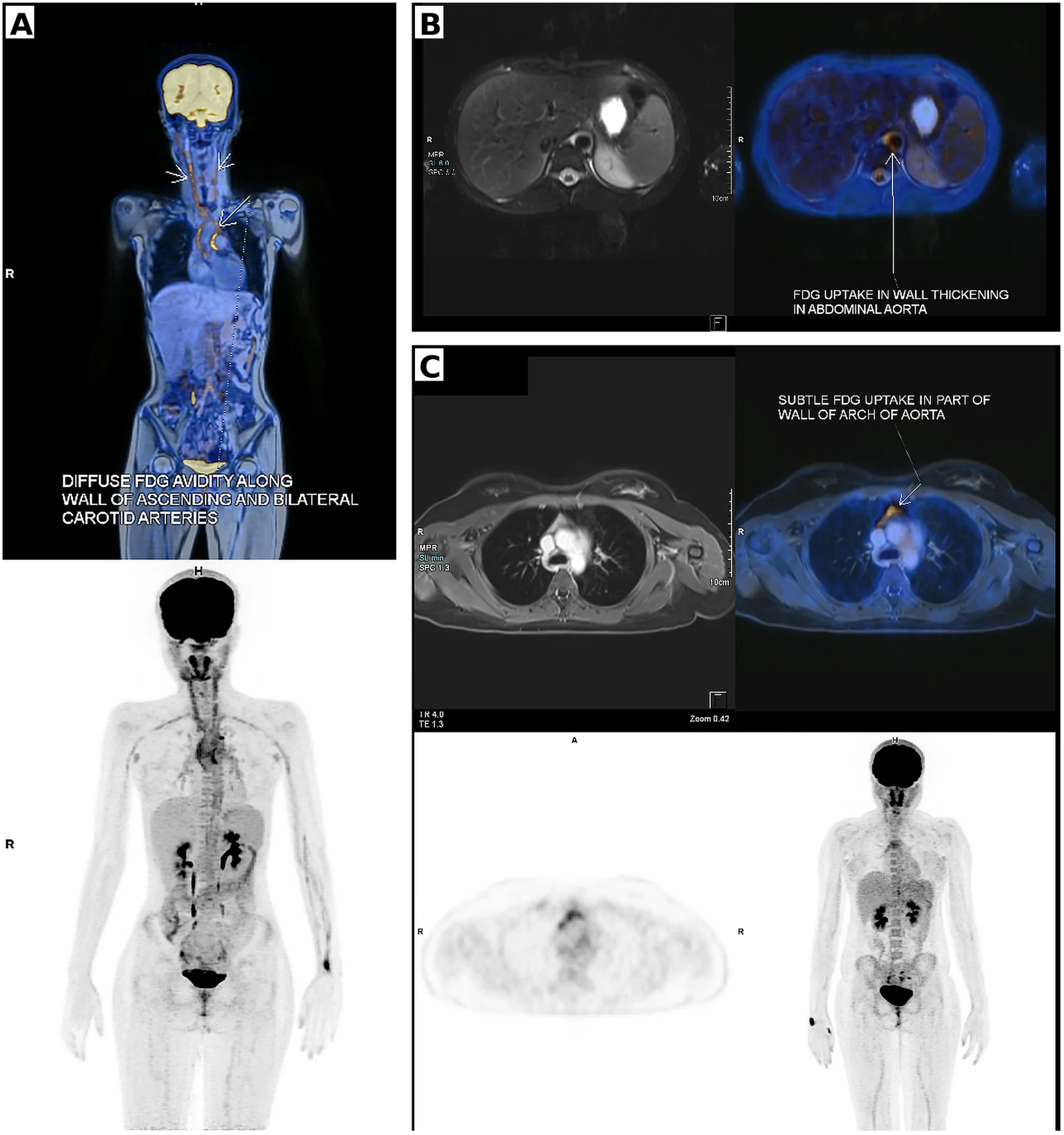
Representative de-identified ^18^F-FDG-PET/MR studies demonstrate metabolically active large-vessel inflammation in children with pre-stenotic Takayasu arteritis presenting with pyrexia of unknown origin. **(A)** Coronal fused ^18^F-FDG-PET/MR (upper) with the corresponding whole-body PET maximum-intensity projection (MIP; lower) shows diffuse FDG-avid wall uptake along the ascending aorta and both common carotid arteries (arrows). **(B)** Axial T2-weighted MR (left) and the corresponding fused axial PET/MR (right) at the level of the upper abdomen in another patient show FDG-avid circumferential wall thickening of the abdominal aorta (arrow). **(C)** Axial MR and fused axial PET/MR (upper) with whole-body PET MIPs (lower) show subtle FDG uptake within the wall of the aortic arch (arrow). In each patient, arterial wall FDG uptake equal to or exceeding hepatic background was taken to indicate active large-vessel vasculitis; all studies were performed before glucocorticoid initiation, and no haemodynamically significant luminal stenosis was present at baseline.

### 3.4 Treatment and follow-up

Following the clinical diagnosis of pre-stenotic Takayasu arteritis, all seven children received induction therapy with pulsed methylprednisolone followed by high-dose oral prednisolone, with mycophenolate mofetil as the first-line steroid-sparing agent in every case. Three children required escalation to the anti-interleukin-6 agent tocilizumab, one of whom additionally received the anti-tumour necrosis factor agent adalimumab.

The cohort was followed for a median of 20 months (range 9–36). Repeat vascular imaging was performed in three children: two, imaged at 20 and 29 months, showed no new stenosis, occlusion, or aneurysm, whereas one child, imaged at 17 months, developed a new short-segment stenosis with intimal thickening of the proximal left subclavian artery, with preserved distal patency. No child developed vascular occlusion, aneurysm, or the classic pulseless features of established Takayasu arteritis during the follow-up period (Table 1; Figure 3).

**Figure 3:**
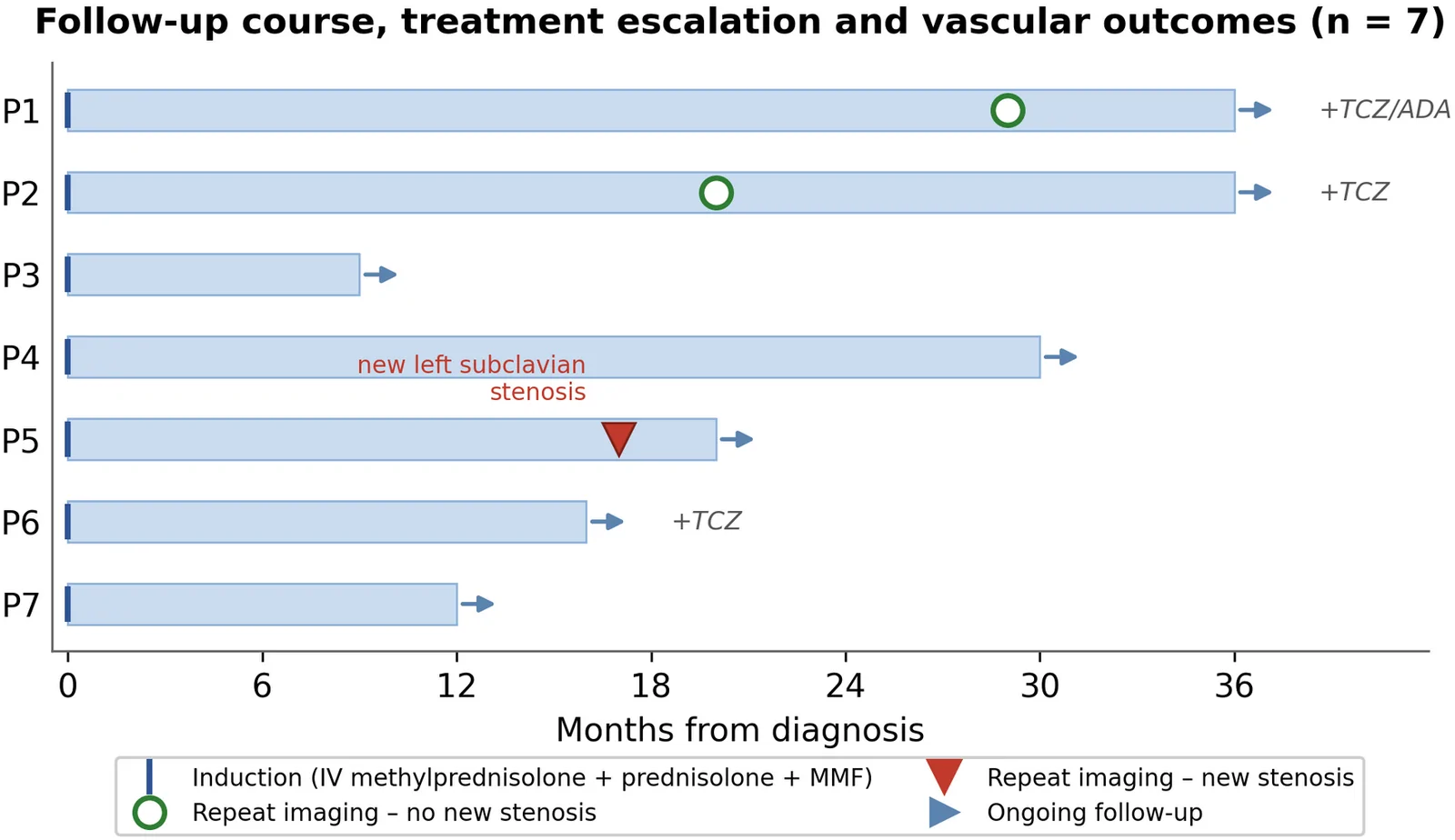
Follow-up course, treatment escalation, and vascular outcomes (*n* = 7). Bars represent follow-up duration from diagnosis; all patients received induction therapy (intravenous methylprednisolone, oral prednisolone, and mycophenolate mofetil) at month 0. Open green circles denote repeat imaging with no new stenosis; the red triangle denotes a new short-segment left subclavian stenosis (Patient 5, 17 months). Arrows indicate ongoing follow-up. TCZ, tocilizumab; ADA, adalimumab.

## 4 Discussion

In this single-centre case series of seven children presenting with PUO, ^18^F-FDG-PET/MR identified metabolically active large-vessel inflammation consistent with pre-stenotic Takayasu arteritis in every case—at a stage when overt vascular signs were largely absent without any structural evidence of flow-limiting stenosis. The combination of an FDG-avid, aortic-predominant distribution, marked systemic inflammation, and the exclusion of infection and malignancy supported a confident diagnosis and enabled early initiation of immunosuppression.

Beyond Takayasu arteritis, ^18^F-FDG-PET/MR contributed to establishing a final diagnosis in 27 of the 28 children with PUO who were scanned (96%), spanning malignancy, histiocytosis, infection, and autoinflammatory disease, with Takayasu arteritis accounting for one quarter—consistent with the recognised role of FDG-PET in fever and inflammation of unknown origin (18, 19). Notably, three of the alternative diagnoses encountered—Erdheim–Chester disease, brucellosis, and tuberculosis—are themselves recognised causes of periaortic or large-vessel FDG uptake and mimics of large-vessel vasculitis (17). This reinforces that a vascular FDG-PET/MR pattern must be interpreted within the clinical context and only after systematic exclusion of infective, histiocytic, and neoplastic causes, as was undertaken in every case in the present series.

The principal advantage of FDG-PET is that it detects the metabolic activity of inflammatory cells infiltrating the arterial wall independently of luminal calibre, allowing recognition of disease during the pre-stenotic phase, when it is most therapeutically responsive (12, 14). This contrasts with structural modalities (MR angiography, CT angiography, and Doppler ultrasonography), which depict the anatomical sequelae of vasculitis and cannot reliably distinguish active inflammatory from chronic fibrotic disease (13). In our cohort, ^18^F-FDG-PET/MR delivered both the metabolic signal and, through its MR angiographic component, co-registered morphological assessment in a single, radiation-sparing examination—an approach that has shown concordance with PET/CT in adults with large-vessel vasculitis and demonstrable added value in childhood-onset Takayasu arteritis (25–27).

Paediatric-specific evidence for FDG-PET in vasculitis remains sparse. The largest prospective paediatric PUO series included only 2 cases of Takayasu arteritis among 110 children (18), and dedicated PET/MR studies in childhood Takayasu arteritis have largely addressed established or treated disease rather than the pre-stenotic, PUO-presenting phase (26, 27). Our series therefore contributes to the small literature by focusing specifically on this earliest and most diagnostically challenging window. Diagnostic delay is characteristic of childhood Takayasu arteritis and permits progression to irreversible vascular remodelling (3, 4), whereas early, active-phase immunosuppression is associated with better vascular outcomes (11). In our cohort, a metabolic diagnosis preceded the classic pulseless phase in all patients and allowed prompt treatment.

Importantly, however, early metabolic diagnosis and treatment did not uniformly prevent structural progression: one child developed a new short-segment left subclavian stenosis at 17 months despite induction and maintenance therapy. This observation underscores that Takayasu arteritis follows a chronic, relapsing–remitting course in which vascular damage may accrue over years (3), and that the absence of stenosis at early follow-up cannot be equated with long-term prevention of complications. Continued serial imaging and disease activity monitoring therefore remain essential even after an early metabolic diagnosis. All seven children satisfied the paediatric EULAR/PRINTO/PRES criteria, in which arterial wall thickening on angiography is a qualifying abnormality, which is relevant to a cohort characterised by wall inflammation rather than established stenosis (21).

The exclusive use of ^18^F-FDG-PET/MR is particularly advantageous in children, coupling metabolic and high-resolution morphological vascular assessment in a single study while avoiding the ionising radiation burden of PET/CT and thereby facilitating the serial imaging that Takayasu arteritis requires (17, 20). Current EULAR recommendations endorse magnetic resonance imaging as the preferred first-line modality in paediatric large-vessel vasculitis, and hybrid PET/MR aligns metabolic activity assessment with this preference (20).

Our study has important limitations. It is a small, single-centre, retrospective case series. Although the 21 children with PUO who underwent ^18^F-FDG-PET/MR and received alternative diagnoses constitute a comparator population, a formal evaluation of diagnostic accuracy was not undertaken as the diagnosis of pre-stenotic Takayasu arteritis incorporated the PET/MR finding (incorporation bias), the vascular PET/MR result was not independently and blindly classified as positive or negative for large-vessel vasculitis across the whole cohort, and no uniform reference standard was applied. Sensitivity, specificity, and predictive values therefore could not be validly derived, and the findings remain hypothesis-generating. FDG-PET/MR was applied selectively to children with persistent PUO and disproportionately elevated inflammatory markers, enriching the cohort for a phenotype likely to yield positive scans (spectrum bias) and probably overestimating the diagnostic yield in an unselected PUO population. Although the follow-up (median 20 months) is longer than in many paediatric series, it remains short relative to the decades-long natural history of Takayasu arteritis, and repeat imaging was not performed in every child. Disease activity was assessed using ITAS-2010, which, although developed and validated in a South Asian population comparable to ours, has limited international adoption and constrains cross-study comparability (23).

Given the small sample size of this preliminary series (*n* = 7), these observations must be regarded as exploratory and hypothesis-generating rather than practice-defining. Prospective, multicentre studies with standardised paediatric PET/MR protocols, harmonised acquisition and interpretation criteria, and long-term follow-up assessing the prevention of structural vascular complications are required before FDG-PET/MR can be recommended for routine integration into paediatric PUO diagnostic algorithms. Future work should also explore emerging tracers (for example, gallium-68-labelled DOTA-D-Phe1-Tyr3-octreotate [68Ga-DOTATATE] and fibroblast-activation-protein inhibitors) and artificial intelligence-based analysis to refine activity assessment and outcome prediction. Within these limits, our series suggests that ^18^F-FDG-PET/MR can identify pre-stenotic childhood Takayasu arteritis in children presenting with PUO, enabling earlier diagnosis and treatment, and supports further investigation of this approach.

## Data Availability

The raw data supporting the conclusions of this article will be made available by the authors, without undue reservation.

## References

[1] Petersdorf RG Beeson PB. Fever of unknown origin: report on 100 cases. Medicine (Baltimore). (1961) 40:1–30. 10.1097/00005792-196102000-00001 13734791

[2] Chow A Robinson JL. Fever of unknown origin in children: a systematic review. World J Pediatr. (2011) 7:5–10. 10.1007/s12519-011-0240-5 21191771

[3] Aeschlimann FA Yeung RSM Laxer RM. An update on childhood-onset Takayasu arteritis. Front Pediatr. (2022) 10:872313. 10.3389/fped.2022.872313 35498790 PMC9043359

[4] Eleftheriou D Varnier G Dolezalova P McMahon AM Al-Obaidi M Brogan PA. Takayasu arteritis in childhood: retrospective experience from a tertiary referral centre in the United Kingdom. Arthritis Res Ther. (2015) 17:36. 10.1186/s13075-015-0545-1 25879697 PMC4392477

[5] Maksimowicz-McKinnon K Clark TM Hoffman GS. Takayasu arteritis and giant cell arteritis: a spectrum within the same disease? Medicine (Baltimore). (2009) 88:221–6. 10.1097/MD.0b013e3181af70c1 19593227

[6] Pugh D Karabayas M Basu N Cid MC Goel R Goodyear CS. Large-vessel vasculitis. Nat Rev Dis Primers. (2021) 7:93. 10.1038/s41572-021-00327-5 PMC9115766 34992251

[7] Rutter M Bowley J Lanyon PC Grainge MJ Pearce FA. A systematic review and meta-analysis of the incidence rate of Takayasu arteritis. Rheumatology (Oxford). (2021) 60:4982–90. 10.1093/rheumatology/keab406 33944899 PMC8566298

[8] Park SJ Kim HJ Park H Hann HJ Kim KH Han S. Incidence, prevalence, mortality and causes of death in Takayasu arteritis in Korea—a nationwide, population-based study. Int J Cardiol. (2017) 235:100–4. 10.1016/j.ijcard.2017.02.086 28283361

[9] Takayasu M. A case with peculiar changes of the central retinal vessels. Acta Soc Ophthalmologicae Japonicae. (1908) 12:554–5.

[10] Russo RA Katsicas MM. Takayasu arteritis. Front Pediatr. (2018) 6:265. 10.3389/fped.2018.00265 30338248 PMC6165863

[11] Hu C Liu L Xiao H Zhao H Chen G Xie Y. Case report: can early full-course tocilizumab therapy reverse vascular stenosis in Takayasu arteritis? Front Immunol. (2025) 16:1593770. 10.3389/fimmu.2025.1593770 40642096 PMC12240941

[12] Tezuka D Maejima Y Sasano T Isobe M. Diagnostic role and feasibility of 18F-FDG PET for assessing Takayasu arteritis: diagnostic efficacy of quantifying 18F-FDG uptake. Ann Nucl Cardiol. (2020) 6:74–80. 10.17996/anc.20-00116 37123485 PMC10133926

[13] Katakura T Shirai T. Positron emission tomography in Takayasu arteritis: a review including patterns of vascular involvement across modalities and regions. J Clin Med. (2025) 14:2939. 10.3390/jcm14092939 40363971 PMC12073023

[14] Kubota R Yamada S Kubota K Ishiwata K Tamahashi N Ido T. Intratumoral distribution of fluorine-18-fluorodeoxyglucose *in vivo*: high accumulation in macrophages and granulation tissues studied by microautoradiography. J Nucl Med. (1992) 33:1972–80. 1432158

[15] Wang ZG Yu MM Han Y Wu FY Yang GJ Li DC. Correlation of Glut-1 and Glut-3 expression with F-18 FDG uptake in pulmonary inflammatory lesions. Medicine (Baltimore). (2016) 95:e5462. 10.1097/MD.0000000000005462 27902598 PMC5134797

[16] Grayson PC Maksimowicz-McKinnon K Clark TM Tomasson G Cuthbertson D Carette S. Distribution of arterial lesions in Takayasu’s arteritis and giant cell arteritis. Ann Rheum Dis. (2012) 71:1329–34. 10.1136/annrheumdis-2011-200795 22328740 PMC3729734

[17] Slart RHJA. FDG-PET/CT(A) imaging in large vessel vasculitis and polymyalgia rheumatica: joint procedural recommendation of the EANM, SNMMI, and the PET Interest Group (PIG), and endorsed by the ASNC. Eur J Nucl Med Mol Imaging. (2018) 45:1250–69. 10.1007/s00259-018-3973-8 29637252 PMC5954002

[18] Pijl JP Kwee TC Legger GE Peters HJH Armbrust W Schölvinck EH. Role of FDG-PET/CT in children with fever of unknown origin. Eur J Nucl Med Mol Imaging. (2020) 47:1596–604. 10.1007/s00259-020-04707-z 32030452 PMC7188721

[19] Hess S Noriega-Álvarez E Leccisotti L Treglia G Albano D Roivainen A. EANM consensus document on the use of [18F]FDG PET/CT in fever and inflammation of unknown origin. Eur J Nucl Med Mol Imaging. (2024) 51:2597–613. 10.1007/s00259-024-06732-8 38676736 PMC11224117

[20] Dejaco C Ramiro S Bond M Bosch P Ponte C Mackie SL. EULAR recommendations for the use of imaging in large vessel vasculitis in clinical practice: 2023 update. Ann Rheum Dis. (2024) 83:741–51. 10.1136/ard-2023-224543 37550004

[21] Ozen S Pistorio A Iusan SM Bakkaloglu A Herlin T Brik R. EULAR/PRINTO/PRES criteria for Henoch-Schönlein purpura, childhood polyarteritis nodosa, childhood Wegener granulomatosis and childhood Takayasu arteritis: Ankara 2008: part II: final classification criteria. Ann Rheum Dis. (2010) 69:798–806. 10.1136/ard.2009.116657 20413568

[22] Craven A Robson J Ponte C Grayson PC Suppiah R Judge A. ACR/EULAR-endorsed study to develop diagnostic and classification criteria for vasculitis (DCVAS). Clin Exp Nephrol. (2013) 17:619–21. 10.1007/s10157-013-0854-0 23996327

[23] Misra R Danda D Rajappa SM Ghosh A Gupta R Mahendranath KM. Development and initial validation of the Indian Takayasu Clinical Activity Score (ITAS2010). Rheumatology (Oxford). (2013) 52:1795–801. 10.1093/rheumatology/ket128 23594468

[24] Blockmans D De Ceuninck L Vanderschueren S Knockaert D Mortelmans L Bobbaers H. Repetitive 18F-fluorodeoxyglucose positron emission tomography in giant cell arteritis: a prospective study of 35 patients. Arthritis Rheum. (2006) 55:131–7. 10.1002/art.21699 16463425

[25] Einspieler I Thürmel K Pyka T Eiber M Wolfram S Moog P. Imaging large vessel vasculitis with fully integrated PET/MRI: a pilot study. Eur J Nucl Med Mol Imaging. (2015) 42:1012–24. 10.1007/s00259-015-3007-8 25876704

[26] Clemente G De Souza AW Leão Filho H Coelho FMA Buchpiguel C Lima M. Does [18F]F-FDG-PET/MRI add metabolic information to magnetic resonance image in childhood-onset Takayasu’s arteritis patients? A multicenter case series. Adv Rheumatol. (2022) 62:28. 10.1186/s42358-022-00260-5 35879743

[27] Clemente G Pereira RMR Aikawa N Silva CA Campos LMA Alves G. Is positron emission tomography/magnetic resonance imaging a reliable tool for detecting vascular activity in treated childhood-onset Takayasu’s arteritis? A multicentre study. Rheumatology (Oxford). (2022) 61:554–62. 10.1093/rheumatology/keab255 33718967

